# 1145. The Role of the Plasmid-Mediated Fluoroquinolone-Resistance (PMFQR) Genes As Resistance Mechanisms in Pediatric Infections due to Enterobacterales (Ent)

**DOI:** 10.1093/ofid/ofab466.1338

**Published:** 2021-12-04

**Authors:** Claire E Pitstick, Steven Marshall, Sreenivas Konda, Rachel L Medernach, T Nicholas Domitrovic, Andrea M Hujer, Nadia K Qureshi, Susan D Rudin, Xiaotian Zheng, Robert A Weinstein, Robert A Bonomo, Latania K Logan

**Affiliations:** 1 Rush University Medical Center, Chicago, Illinois; 2 Louis Stokes Cleveland Medical Center, Cleveland, OH; 3 University of Illinois at Chicago, Chicago, Illinois; 4 VA Cleveland Medical Center, Cleveland, OH; 5 Loyola University Medical Center, Maywood, IL; 6 Northwestern University Feinberg School of Medicine; Ann and Robert H. Lurie Children’s Hospital of Chicago, Chicago, IL; 7 Louis Stokes Cleveland VA Medical Center, Cleveland, OH

## Abstract

**Background:**

Fluoroquinolones (FQs) are not commonly prescribed in children, yet the increasing incidence of multidrug resistant (MDR) Ent infections in this population often reveals FQ resistance. We sought to define the role of FQ resistance in the epidemiology of MDR Ent in children, with an overall goal to devise treatment and prevention strategies.

**Methods:**

A case-control study of children (0-18 years) at 3 Chicago hospitals was performed. Cases had infections by FQ susceptible, 3^rd^ generation cephalosporin-resistant (3GCR) and/or carbapenem-resistant (CR) Ent harboring a non or low level expressed PMFQR gene (PMFQS Ent). Controls had FQR infections due to 3GCR and/or CR Ent with expressed PMFQR genes (PMFQR Ent). We sought *bla* genes by PCR or DNA (BD Max Check-Points assay®) and PMFQR genes by PCR. We performed Rep-PCR, MLST, and *E. coli* phylogenetic grouping. Demographics; comorbidities; and device, antibiotic, and healthcare exposures were evaluated. Predictors of infection were assessed.

**Results:**

Of 170 G3CR and/or CR Ent isolates, 85 (50%) were FQS; 23 (27%) had PMFQR genes (PMFQS cases). 85 (50%) were FQR; 53 (62%) had PMFQR genes (PMFQR controls). The median age for children with PMFQS Ent and PMFQR Ent were 4.3 and 6.2 years, respectively (p=NS). Of 23 PMFQS Ent, 53% were *Klebsiella* and of 53 PMFQR Ent, 76% were *E. coli*. The most common *bla* and PMFQR genes in PMFQS Ent were *bla*_SHV ESBL_ (44%); *oqxB* (57%) and *aac-6’1b-cr* (52%) and in PMFQR Ent were *bla*_CTX-M-1 group_ (76%); *aac-6’1b-cr* (91%) and *oqxA* (17%).

Multivariable regression analysis showed children with PMFQS Ent infections were more likely to have hospital onset infection (OR 5.7, 95% CI 1.6-22) and isolates with multiple *bla* genes (OR 3.8, 95% CI 1.1-14.5). The presence of invasive devices mediated the effects of healthcare setting in the final model. Differences in demographics, comorbidities, or antibiotic use were not found.

**Conclusion:**

Paradoxically, PMFQS Ent infections were often hospital onset and PMFQR Ent infections were community onset. PMFQS Ent commonly co-harbored multiple *bla* and PMFQR genes, affecting therapeutic options and suggesting need for contact precautions. Control of PMFQS *Ent* infections in children will require validating sources and risk factors.

**Disclosures:**

**Robert A. Bonomo, MD**, **entasis** (Research Grant or Support)**Merck** (Grant/Research Support)**NIH** (Grant/Research Support)**VA Merit Award** (Grant/Research Support)**VenatoRx** (Grant/Research Support)

